# Insights From Michael Polanyi: Tacit Knowledge and Its Critical Importance in Medical Education

**DOI:** 10.7759/cureus.102205

**Published:** 2026-01-24

**Authors:** Thomas J Papadimos, Justin Hsu, Scott M Pappada

**Affiliations:** 1 Anesthesiology, Surgery, and Critical Care, The University of Toledo College of Medicine and Life Sciences, Toledo, USA; 2 Surgery, The University of Toledo College of Medicine and Life Sciences, Toledo, USA; 3 Anesthesiology, The University of Toledo College of Medicine and Life Sciences, Toledo, USA; 4 Bioengineering, The University of Toledo College of Engineering, Toledo, USA

**Keywords:** educational philosophy, machine learning (ml), medical resident education, medical school education, philosophy of medicine

## Abstract

With the onset of the Fourth Industrial Revolution (the digital age), medical education is undergoing dramatic change. Artificial intelligence (AI) and machine learning (ML) have driven an increase in virtual learning sessions and meetings. Bedside teaching is increasingly at risk of becoming an endangered mode of education. Tacit knowledge, as defined and explained by Michael Polanyi, relies heavily on direct interaction with a teacher and mentor. The question, therefore, arises, “Can artificial intelligence and machine learning function as meaningful adjuncts that support the transfer of tacit knowledge in medical education, or will bedside teaching ultimately be replaced entirely by machine learning and artificial intelligence?”

## Editorial

With the onset of the Fourth Industrial Revolution (the digital age) [1] and the profound impact of the coronavirus disease 2019 (COVID-19) pandemic on medical education, particularly through its acceleration of machine learning (ML) and artificial intelligence (AI), traditional bedside teaching in medicine may be increasingly threatened. The erosion of this close educational relationship endangers the transfer of tacit knowledge from teacher to learner.

Michael Polanyi, a Hungarian-British physical chemist who later shifted his academic focus to economics and philosophy, coined the terms “tacit knowing” and “tacit knowledge [2].” Polanyi sought to understand why “our whole civilization was pervaded by the dissonance of an extreme critical lucidity and an intense moral conscience [2],” and undertook an inquiry to identify the “root of this condition [2].” This intellectual pursuit led him to examine the nature of knowledge, and he ultimately concluded that “we can know more than we tell [2].” He argued that teachers, mentors, practitioners, and others who hold expertise are often unaware of the full scope of their knowledge when they perform a task, carry out a procedure, or make a diagnosis. To pass this knowledge on to a learner, trust and sustained close personal interaction with a teacher or mentor are essential. Polanyi’s now rarely cited books, Personal Knowledge (1958) and The Tacit Dimension (1966), emphasize the fundamental insight that “we know more than we can tell [2],” and underscore that the transmission of such knowledge, which is often difficult to articulate or explain, is critically important for enabling learners to perform tasks effectively, appropriately, and safely [3,4].

Tacit knowledge is critically important in the medical education of all learners, including students, residents, and allied health professionals. Tacit knowledge is also known as implicit knowledge. It represents a type of understanding that is difficult to communicate through writing or speech. It relates to “know-how” rather than “know-what [2,5]”. This encompasses insights, wisdom, experiential understanding, intuition, and motor skills. Classic examples include recognizing a face, riding a bicycle, or interpreting a nonverbal cue indicating another person’s response in a specific situation. This contrasts with explicit knowledge, which can be conveyed through writing, textbooks, manuals, documents, videos, and similar resources. In other words, a learner who is not physically present with their teacher or mentor when examining a patient or performing a procedure is highly likely to fail in replicating that specific task or skill. “Tacit knowing matters; it is the basis of practical competence [6].”

Why present this brief, poignant exposition about tacit learning and knowledge? The Fourth Industrial Revolution is already here, bringing Zoom sessions, remote classrooms, and online lectures, along with the growing influence of ML and AI. It is extremely important, even during the preclinical years, for learners to maintain close interaction with teachers and mentors to benefit from tacit knowing and tacit knowledge, which includes wisdom, experience, insights, and intuition that can be acquired from a teacher or mentor but cannot be formally codified, structured, or fully articulated. “Even though guidelines and codifications can play a practical role in informing clinical science, they rest on a body of tacit or implicit skill that is, in principle, ineliminable. It forms the bedrock of good judgement and unites the integration of research, expertise, and values [7].” The fact remains that, “Along with the use of existing medical rules, there is a separate channel that physicians rely on when making their decisions: their intuition [8].”

We must acknowledge that tacit knowledge, by its very nature, lacks the objectivity and reproducibility that underpin evidence-based medicine (EBM). However, tacit knowledge is critical for applying the best evidence through clinical competence and experience tailored to the patient and their values. EBM is codified and explicit, but clinical medicine demands a patient-centered approach. Tacit knowledge can assist physicians not only in applying EBM but also in navigating the ethics and humanism of each clinical situation. We do not view EBM and tacit knowledge as competing camps, but as strengths that must be thoughtfully and carefully integrated into medical education.

The difficulty and the truth of the matter is that digital and remote education are here to stay. The clinical bonding between the teacher or mentor and the learner has changed from what it was at the end of the last century and the beginning of this one. While having the learner at the bedside is an ideal way to educate them in how a clinician thinks, diagnoses, prognosticates, and approaches the patient and their family, an alternative mode of supplementary education may be necessary and is, in fact, available.

Investigators believe the answer may lie in high-fidelity medical simulation, which can occur in the presence of a competent clinician in the right kind of learning environment equipped with advanced digital capabilities. While the aforementioned concerns about the risk to tacit knowledge and tacit learning/knowing are palpable, concerned medical providers and scientists are actively creating and implementing advanced platforms using ML and AI to address this situation [9]. These platforms allow training 24 hours a day and can track and analyze behavioral, cognitive, and physiological factors that are critical for assessing and instructing learners. Many of these large language ML/AI models essentially attempt to replicate the teacher/mentor relationship, guiding learners through the learning process and even predicting when learners will require retraining. Such emerging platforms leverage AI to maintain a dynamic “pulse” on learners throughout their simulated clinical experiences by integrating multimodal streams of data, including verbal utterances, team communication, patient state changes from simulated health records and human patient simulators, and learners’ physiologic data into a unified analytic framework that provides real-time, data-driven feedback to complement instructors [10].

To provide a complete picture of the learner experience, current and future platforms must integrate audio, video, simulator logs, and performance checklists to generate visualizations and metrics that reveal how teams coordinate, how individuals respond to evolving patient conditions, and where cognitive overload or lapses in nontechnical skills may occur, thereby capturing subtleties in communication, timing, and workload that even experienced human clinical faculty might miss. Emerging technologies like these have broader implications for real-world patient care. When extended to clinical settings through continuous monitoring via wearables on healthcare professionals, these platforms can track provider stress [11], cognitive load, and real-time decision-making, in combination with electronic health records, and exert a significant influence on medical education and patient care. These technologies act as a second set of eyes and ears to support richer debriefings and targeted coaching, and they aim to transmit tacit elements such as intuition and contextual judgment without replacing the irreplaceable human mentorship.

In medical training, where learning is often epitomized by the adage “see one, do one, teach one,” AI-based simulations and intraoperative adjuncts are being developed that have the potential to redefine how technical skills are traditionally taught. Technical skills training has become increasingly difficult in the modern age, constrained by duty-hour restrictions and compounded by growing pressures for productivity and efficiency, as well as medicolegal scrutiny. AI in surgical education offers the potential to alleviate these challenges by delivering customized feedback and individualized learning, whether through virtual or augmented reality medical simulation, automated video review, or a virtual operative assistant [12]. In the operating room, advances in AI and computer vision leverage the collective knowledge of experienced surgeons to provide real-time intraoperative guidance, helping to identify uncertain surgical anatomy [13].

The learning impact of these platforms is clear and highly valuable: they can record training sessions visually, transcribe a learner’s words and responses using AI mapping, determine whether an answer is correct, and even analyze physiological responses such as heart rate, respiration, and perspiration. These systems and tools are crucial to education and training and will become even more essential in the coming decade.

Nonetheless, the overall efficacy of supplementing medical education through simulation requires extensive evaluation, particularly regarding the transfer of tacit knowledge. It is of critical importance to effectively preserve the transfer of tacit knowledge from teacher to learner. The digital age, with its advancements in ML and AI, is here to stay and can serve as a valuable adjunct to the transfer of tacit knowledge. The question arises: can these ML/AI models and platforms establish a meaningful bond with learners? Can they convey clinical nuances to learners? Can they influence learner motivation and attitude? Current models cannot fully replace an instructor. However, there may come a time in the future when a very human-looking robot, speaking in a human-like voice, with manual dexterity surpassing that of humans, and an AI brain containing the clinical nuances of thousands of instructors, could be offered as an alternative or even replacement for human teachers and mentors.

Careful thought must be given to the future approach to medical education because it is very personal, requires professional intimacy, may be limited or hindered by technology, and by learners’ attitudes (Generation Z has a different way of approaching their concerns) [14]. “Tacit knowing is the unifying factor in evidence-based medicine and clinical judgement [7].” This will be a challenging task for AI to master, and it may be a task that is neither possible nor desirable. Only time will tell. According to 16th-century medical lore, skilled practitioners need to possess “the hands of a woman, the eyes of an eagle, and the heart of a lion [15].” This truism still holds in this digital age. An AI robot will probably be able to perform the first two requisites, but the last one…the heart of a lion? We think not; that will always be left to humans and lions. Michael Polanyi would agree.
